# Nomogram predicts atrial fibrillation after coronary artery bypass grafting

**DOI:** 10.1186/s12872-022-02824-1

**Published:** 2022-08-30

**Authors:** Jingshuai Gong, Yangyan Wei, Qian Zhang, Jiwen Tang, Qing Chang

**Affiliations:** Department of Cardiovascular Surgery, The Affiliated Hospital of Qingdao University, 16 Jiangsu Road, Qingdao, 266003 China

**Keywords:** Coronary artery bypass grafting, Postoperative atrial fibrillation, Nomogram

## Abstract

**Objective:**

Using the nomogram to intuitively predict atrial fibrillation after coronary artery bypass grafting. Identify high-risk patients with atrial fibrillation and provide preoperative protective therapy.

**Methods:**

A total of 397 patients that underwent coronary artery bypass grafting were consecutively enrolled. Independent predictors of patients were analyzed by multivariate logistic regression. Two nomograms were constructed to predict postoperative atrial fibrillation.

**Results:**

The incidence of postoperative atrial fibrillation in this study was 29% (115/397). Multivariate Logistic showed that Age, Operative Time > 4 h, Left Atrial Diameter > 40 mm, Mean Arterial Pressure, Body Mass Index > 23 kg/m^2^, Insulins, and Statins were independently associated with atrial fibrillation after isolated coronary artery bypass grafting. The nomogram of postoperative atrial fibrillation in patients was constructed using total predictor variables (AUC = 0.727, 95% CI 0.673–0.781). The model was internally validated (AUC = 0.701) by K-fold Cross-validation resampling (K = 5, Times = 400). To make an early intervention, the intraoperative information of the patients was excluded. Only 6 variables before surgery were used to establish the brief nomogram to predict postoperative atrial fibrillation (AUC = 0.707, 95% CI 0.651–0.764). The brief model was internally validated (AUC = 0.683) by resampling with K-fold Cross-validation resampling.

**Conclusions:**

These two nomograms could be used to predict patients at high risk for atrial fibrillation after isolated coronary artery bypass grafting.

## Introduction

Postoperative atrial fibrillation (POAF) is the most common complication after cardiac surgery [1]. The incidence of new-onset POAF after coronary artery bypass grafting (CABG) is 20–40% [2–4]. POAF usually occurs 2–4 days after CABG [5]. The occurrence of POAF will increase the risk of other complications, prolong the hospital stay, and increase the economic burden on patients [6]. POAF is a complex pathophysiological mechanism, and the exact pathophysiological mechanism has not been fully elucidated [7–9]. But, there is a lot of evidence that POAF may be associated with inflammation, myocardial ischemia, sympathetic activation, etc.[1, 5] Overall, the pathogenesis of POAF can be classified into acute factors induced by surgical intervention and chronic factors related to structural heart disease and aging of the heart [10].

Currently, many studies have raised the importance of preventing patients from developing POAF and have proposed their predictive models [11–13]. However, no nomogram was used to predict the POAF. After the start of our trial, a paper using nomograms to predict POAF was published [14]. But, Fan et al. have some deficiencies [14]. Based on previous studies, this study will propose two new nomograms.

## Patients and methods

### Study population

**Fig. 1:**
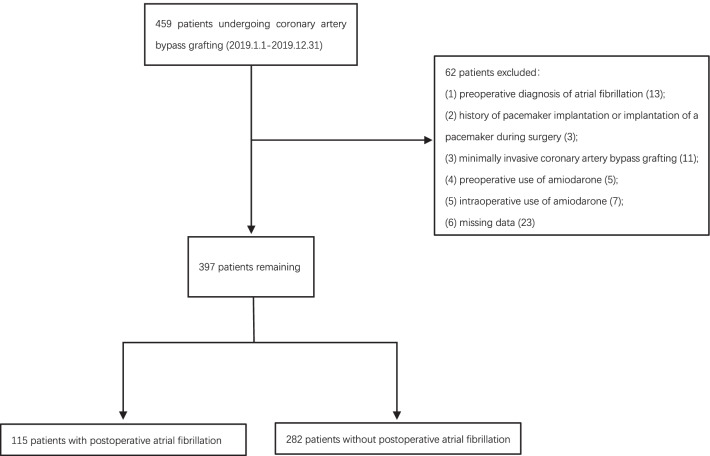
Flow chart of patient selection for the present analysis

This study was a cohort study of patients who underwent CABG at the Affiliated Hospital of Qingdao University from January 2019 to December 2019. The cardiac center is a tertiary referral center and all procedures are performed independently by experienced physicians. The retrospective study did not require informed consent from the Ethics Committee of the Affiliated Hospital of Qingdao University. The inclusion criteria were patients older than 18 years and patients who underwent isolated CABG. The exclusion criteria were as follows: (1) Preoperative diagnosis of atrial fibrillation (AF); (2) History of pacemaker implantation or intraoperative implantation of a pacemaker; (3) Minimally invasive CABG; (4) Preoperative and intraoperative use of amiodarone; (5) Missing data (Fig. 1). Ultimately, 397 patients were screened for analysis in this study.

### Diagnosis and definitions

In this study, perioperative data for each patient were retrospectively collected. The preoperative laboratory data of all patients were the last recorded data before surgery.

Emergency operative is defined as surgery performed within 48 h of admission to the hospital. Palpitations are defined as uncomfortable feelings of the abnormal beating of the heart. POAF is defined as patients who were in sinus rhythm before CABG but developed new-onset AF after CABG. AF was defined as an irregular heart rhythm without repetitive patterns and prominent P waves for at least 30 s. In this study, each patient underwent continuous electrocardiogram (ECG) monitoring over 7 days. Patients were observed for new-onset atrial fibrillation up to 7 days after CABG. The estimated glomerular filtration rate (eGFR) was calculated using the Chronic Kidney Disease Epidemiology Collaboration (CKD-EPI) equation: eGFR = α × (Scr/β)γ × 0.993age,if patient is female: α = 144, β = 62, γ = − 0.329(Scr ≤ 62umol/L) or − 1.209(Scr > 62umol/L); if patient is male: α = 141, β = 80, γ = − 0.411(Scr ≤ 80umol/L) or − 1.209(Scr > 80umol/L).

### Perioperative period management

In this study, all 397 patients underwent isolated CABG. The patients had not received any arrhythmia treatment prior to CABG. All patients underwent surgery using the same anesthetic drugs and surgical techniques. During the operation, firstly, the left internal mammary artery can be used to bypass the anterior descending branch, or the diagonal branch and other blood vessels can be anastomosed sequentially according to the condition. Second, great saphenous vein graft for other diseased vessels. After surgery, the patient is closely observed and documented with ECG monitoring.

### Statistical analysis

The normally distributed measurement data is expressed as mean ± standard deviation (X ± SD), and the non-normally distributed measurement data is expressed as the median (25th, 75th percentile). Univariate logistic regression analysis was used to explore potential predictors of postoperative AF. Potential predictors of *P* < 0.05 were included in multivariate logistic regression and modeled using the backward stepwise method. A nomogram was constructed, the model discrimination was verified and the nomogram was internally validated by K-fold Cross-validation (K = 5, Times = 400) resampling, and the resampled AUC was calculated. The calibration degree, the clinical applicability of the model, and the importance of each index of the model were analyzed. Deleting the intraoperative data and repeating the above steps to model again. A two-tailed, *P* < 0.05 was defined as a statistically significant difference. Data analysis was performed using SPSS software version 26.0 and R software version 4.1.2.

## Results

### Patient characteristics

From January 2019 to December 2019, 459 isolated CABG patients were consecutively enrolled in the study. According to the exclusion criteria, finally, a total of 397 patients were included in the final analysis. Baseline characteristics of both with POAF group and without POAF group (Table 1). The age of the POAF group was 67.3 ± 7.9 years, and 84(73.0%) were male patients, 71 (61.7%) patients with hypertension, 46 (40.0%) patients with diabetes, and 13 (11.3%) patients undergoing on-pump surgery. The age without POAF group was 63.2 ± 7.7 years, and 199 (70.6%) were male patients, 71 (61.7%) patients with hypertension, 105 (37.2%) patients with diabetes, and 24 (8.5%) patients undergoing on-pump surgery.

**Table 1:** Baseline characteristics

Variables	Without POAF (n = 282)	With POAF (n = 115)	*P* value
*Demographics*			
Age, years*	63.2 ± 7.7	67.3 ± 7.9	< 0.001
Male	199 (70.6%)	84 (73.0%)	0.710
Heart rate, beats/min	69.5 ± 10.4	70.1 ± 11.5	0.629
MAP, mmHg*	90.3 ± 10.4	87.7 ± 11.4	0.025
BMI > 23, kg/m^2^*	226 (80.1%)	81 (70.4%)	0.037
*Social history*			
Current smoker	115 (40.8%)	48 (41.7%)	0.949
Alcohol	77 (27.3)	29 (25.2)	0.763
*Medical history*			
Diabetes	105 (37.2%)	46 (40.0%)	0.688
Hypertension	185 (65.6%)	71 (61.7%)	0.539
PVDs	10 (3.5%)	3 (2.6%)	0.869
Stroke	34 (12.1%)	18 (15.7%)	0.424
COPD	3 (1.1%)	3 (2.6%)	0.490
CKD	4 (1.4%)	5 (4.3%)	0.159
*Cardiac disease history*			
Palpitate	34 (12.1%)	19 (16.5%)	0.306
Heart failure	16 (5.7%)	9 (7.8%)	0.567
ACS*	267 (94.7%)	115 (100.0%)	0.026
OMI	19 (6.7%)	12 (10.4%)	0.299
Stent	40 (14.2%)	13 (11.3%)	0.547
LAD > 40,mm*	175 (62.1%)	86 (74.8%)	0.021
Abnormal wall movement	179 (63.5%)	85 (73.9%)	0.060
LVEF, %*	57.8 ± 7.1	55.1 ± 8.5	0.001
*Medications*			
ACEI	39 (13.8%)	10 (8.7%)	0.214
ARB*	98 (34.8%)	26 (22.6%)	0.025
BRB	193 (68.4%)	67 (58.3%)	0.069
CCB	110 (39.0%)	34 (29.6%)	0.097
Diuretics	109 (38.7%)	51 (44.3%)	0.349
Statins*	222 (78.7%)	71 (61.7%)	0.001
Insulins*	123 (43.6%)	31 (27.0%)	0.003
OHAs	85 (30.1%)	31 (27.0%)	0.609
Cardiac inotropic drugs	26 (9.2%)	13 (11.3%)	0.655
*Preoperative laboratory parameters*			
Hemoglobin, g/L	130.7 ± 17.2	128.1 ± 20.1	0.201
White blood cell count, 10^9^/L	6.6 ± 1.9	6.6 ± 2.0	0.852
Neutrophil count, 10^9^/L	4.1 ± 1.6	4.1 ± 1.6	0.924
Monocyte count, 10^9^/L	0.5 ± 0.3	0.5 ± 0.2	0.542
Lymphocyte count, 10^9^/L	1.9 ± 0.6	1.7 ± 0.7	0.052
Platelet count, 10^9^/L*	224.6 ± 59.6	211.2 ± 60.6	0.044
Uric acid, µmol/L	342.3 ± 97.9	362.2 ± 105.6	0.074
Serum creatinine, µmol/L	78.1 [65.0, 93.2]	81.0 [65.0, 95.8]	0.743
eGFR, mL/min/1.73m^2^	81.9 ± 20.9	78.2 ± 22.8	0.125
Triglyceride, mmol/L	1.4 [1.1, 2.0]	1.4 [1.0, 1.9]	0.369
LDL, mmol/L	2.5 ± 0.9	2.6 ± 0.9	0.781
Glucose, mmol/L	5.5 [4.8, 6.8]	5.4 [4.9, 7.2]	0.750
Albumin, g/L*	45.6 ± 8.7	43.1 ± 7.7	0.009
Prealbumin	255.1 ± 66.6	257.8 ± 57.9	0.702
Kalium, mmol/L	4.2 ± 0.4	4.2 ± 0.4	0.708
*Surgical information*			
Emergency surgery	3 (1.1%)	0 (0.0%)	0.637
Preoperative IABP*	18 (6.4%)	15 (13.0%)	0.048
On-pump surgery	24 (8.5%)	13 (11.3%)	0.498
Left internal mammary artery	238 (84.4%)	87 (75.7%)	0.056
Left anterior descending	270 (95.7%)	112 (97.4%)	0.624
Diagonal branch	183 (64.9%)	75 (65.2%)	1.000
Obtuse marginal artery	156 (55.3%)	60 (52.2%)	0.646
Posterior descending artery	167 (59.2%)	68 (59.1%)	1.000
Left posterior artery	25 (8.9%)	10 (8.7%)	1.000
Incomplete revascularization	4 (1.4%)	2 (1.7%)	1.000
Operative time > 4 h,min*	118 (41.8%)	65 (56.5%)	0.011

MAP, Mean arterial pressure; BMI, Body mass index; PVDs, peripheral vascular diseases; COPD, Chronic obstructive pulmonary disease; CKD, Chronic Kidney Disease; ACS, Acute Coronary Syndromes; OMI, Old myocardial infarction; LAD, Left atrial diameter; LVEF, Left Ventricular ejection fraction; ACEI, Angiotensin Converting Enzyme Inhibitors; ARB, Angiotensin Receptor Blockers; BRB,β-receptor blockers; CCB, Calcium channel blockers; OHAs, Oral hypoglycemic agents; eGFR, estimated glomerular filtration rate; LDL, Low density lipoprotein; Preoperative IABP, Preoperative intra-aortic balloon pump

*P < 0.05

### Predictors of postoperative atrial fibrillation

The data collected showed the incidence of POAF: 28.9% (115/397). POAF may be associated with 13 variables: Age, Mean Arterial Pressure (MAP), Body Mass Index (BMI) > 23 kg/m^2^, Acute Coronary Syndrome, Albumin, Platelet count, Left Ventricular Ejection Fraction (LVEF), Left Atrial Diameter (LAD) > 40 mm, Insulins, Angiotensin Receptor Blockers, Statins, Preoperative Intra-aortic Balloon Pump and Operative Time > 4 h (h) by univariate analysis (*P* < 0.05). Then, using logistic stepwise backward regression analysis, the results showed that 7 variables: Age, Operation Time > 4 h, LAD > 40 mm, MAP, BMI > 23 kg/m^2^, Insulins, and Statins were independent predictors of AF after CABG (*P* < 0.05; Table 2).

**Table 2:** Multivariable logistic regression analysis of 7 variables

Variables	Exp(B)	95% CI		P
Age, years	1.057	1.023	1.092	0.001
Operative time > 4 h, min	1.935	1.199	3.122	0.007
Insulins	0.544	0.324	0.913	0.021
Statins	0.551	0.326	0.93	0.026
LAD > 40, mm	1.879	1.062	3.323	0.03
MAP, mmHg	0.976	0.954	0.998	0.032
BMI > 23, kg/m^2^	0.549	0.308	0.979	0.042

Abbreviations as in Table 1

### The establishment and verification of the predicted nomogram

**Fig. 2:**
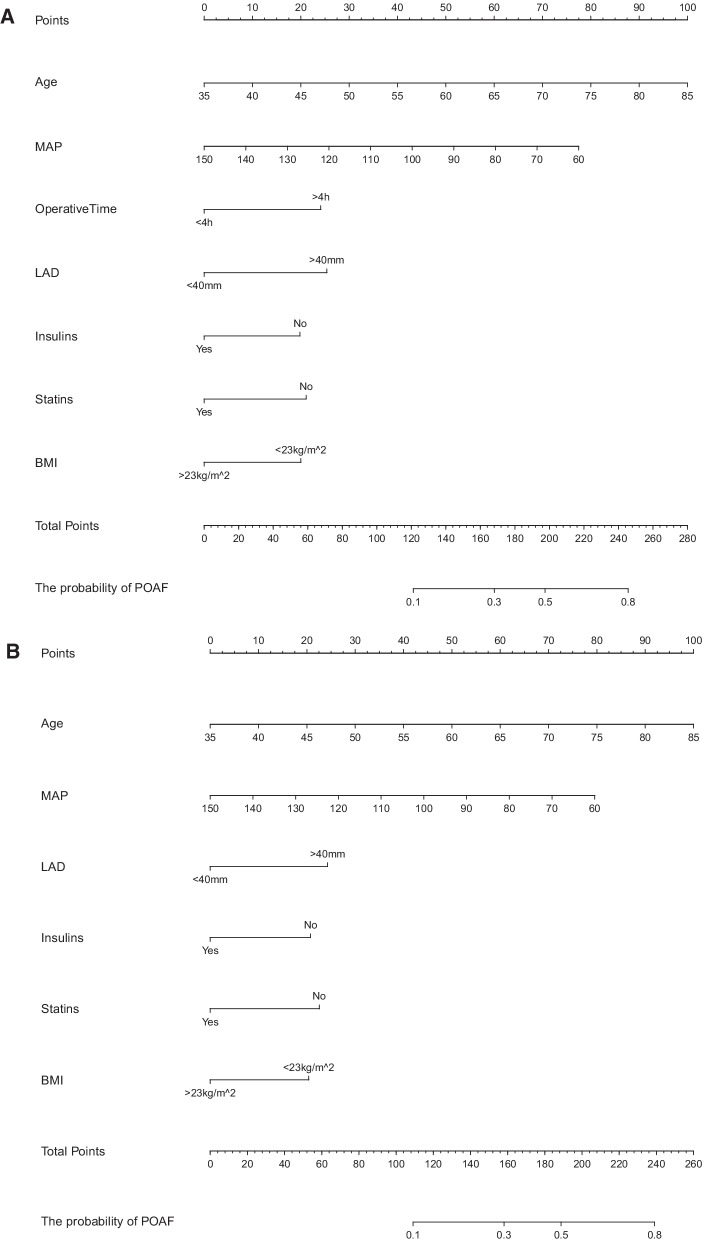
**A** The nomogram for predicting new-onset postoperative atrial fibrillation following isolated coronary artery bypass grafting. **B** The brief nomogram for predicting new-onset postoperative atrial fibrillation following isolated coronary artery bypass grafting

**Fig. 3:**
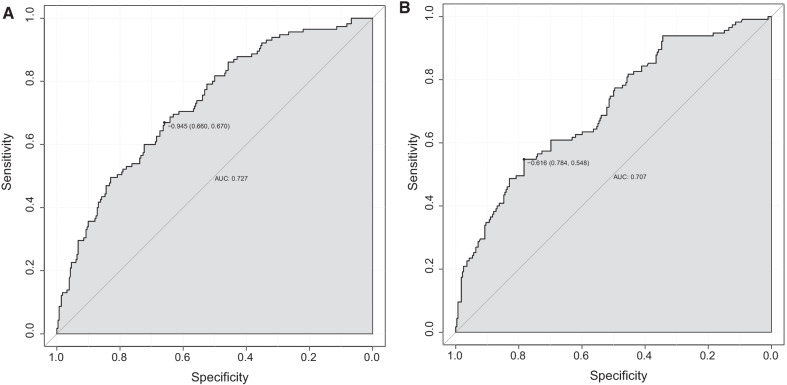
**A** ROC curve of 7 Predictors, **B** ROC curve of 6 Predictors

**Fig. 4:**
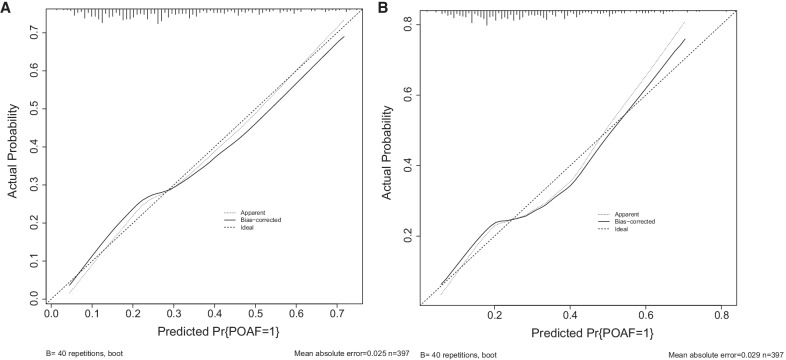
**A** The calibration curve of 7 Predictors, **B** The calibration curve of 6 Predictors

**Fig. 5:**
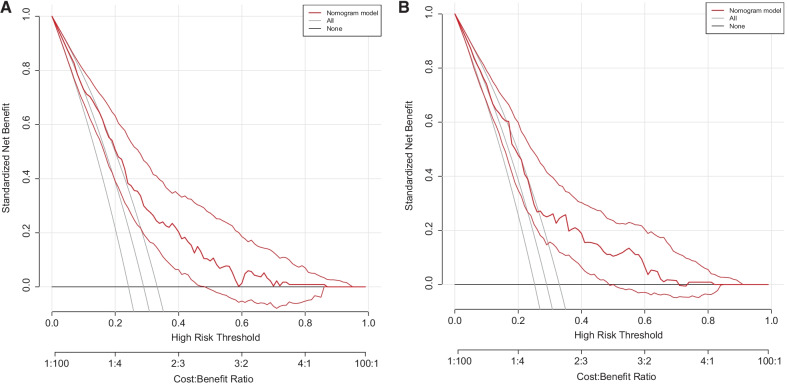
**A** DCA of 7 Predictors, **B** DCA of 6 Predictors

**Fig. 6:**
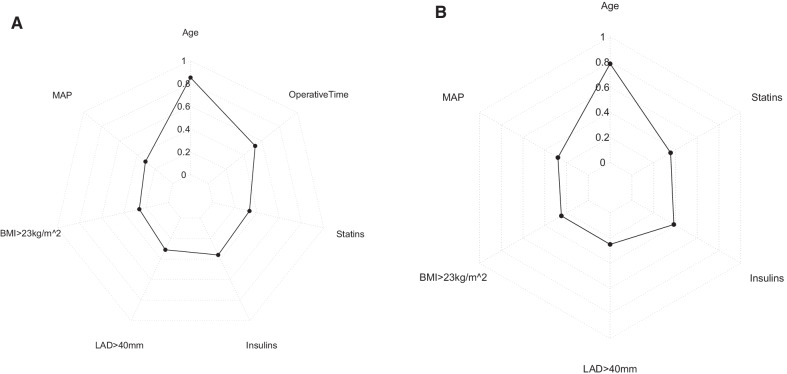
**A** Radar Chart of 7 Predictors, **B** Radar Chart 6 of Predictors

The nomogram was constructed using the 7 independent predictors described above to predict the risk of POAF in CABG (Fig. 2A). The nomogram assigns 7 variables to the patient and adds up the assignments of each variable of the patient to obtain the probability of postoperative AF in the patient. The discriminant degree of the prediction model was established by the ROC curve test (AUC = 0.727, 95% CI 0.673–0.781; Fig. 3A). Models were internally validated (AUC = 0.701) by resampling with K-fold Cross-validation (K = 5, Times = 400). The calibration curve of the model shows a good agreement between the predicted and observed probabilities of the nomogram (Fig. 4A). The Decision Curve Analysis (DCA) red curve represents the clinical benefit of patients with different AF risk levels (Fig. 5A). This suggests that the use of this model to identify postoperative AF for intervention may provide more benefit than the original treatment strategy. Building the radar chart to identify the importance of 7 predictor variables (Fig. 6A). In addition, by calculating the total score of all patients, it was analyzed that when the total score of patients was greater than or equal to 165, the risk of POAF was high. Only preoperative variables were analyzed using logistic stepwise backward regression (Table 3). The brief nomogram was established to predict POAF, including only 6 preoperative data: Age, LAD > 40 mm, MAP, BMI > 23 kg/m^2^, Insulins, and Statins (Fig. 2B). Establishing the ROC curve to test the discrimination of the brief prediction model (AUC = 0.707, 95% CI 0.651–0.764; Fig. 3B). Models were internally validated (AUC = 0.683) by K-fold Cross-validation resampling (K = 5, Times = 400). The calibration curve of the brief model (Fig. 4B). The DCA curve of the brief model (Fig. 5B). Building the radar chart to identify the importance of 6 predictor variables (Fig. 6B). In last, by calculating the total score of all patients, it was analyzed that when the total score of patients was greater than or equal to 166, the risk of POAF was high.

**Table 3:** Multivariable logistic regression analysis of 6 variables

Variables	Exp(B)	95% CI		P
Age, years	1.055	1.021	1.089	0.001
Insulin	0.538	0.322	0.898	0.018
Statin	0.546	0.326	0.915	0.022
MAP, mmHg	0.977	0.955	0.999	0.037
LAD > 40, mm	1.774	1.012	3.111	0.045
BMI > 23, kg/m^2^	0.56	0.317	0.99	0.046

Abbreviations as in Table 1

## Discussion

The incidence of POAF in our study was 29%, which is consistent with the 20–40% incidence of POAF in previous studies with CABG. [2–4] It is also similar to the 28% incidence of POAF reported by Fan et al. in a Chinese population-based study.[14]Compared with the study by Fan et al. that collected patient baseline data [14]. The independent predictors of POAF in patients with isolated CABG were analyzed after the trial began by collecting more granular patient baseline information. Finally, Age, Insulins, Statins, LAD > 40 mm, MAP, BMI > 23 kg/m^2^, and intraoperative Operative Time > 4 h were determined. By using 7 variables to make a POAF prediction model, it shows higher accuracy than established prediction models, [12–14] and these variables are more readily available clinically. Although the 7 variables model (AUC = 0.727) showed good performance, it contained an intraoperative piece of information that made this model unable to differentiate and intervene preoperatively. More and more studies have proved that if POAF can be prevented early, the occurrence of short-term and long-term complications after surgery can be reduced. [6, 15, 16] Some studies have also suggested that preoperative β-blocker drugs treatment [17], intraoperative right posterior pericardiotomy [6], etc. are all beneficial to reducing the occurrence of POAF in patients. Therefore, we tried to remove the intraoperative information in the model, only used the preoperative information, and then remodeled it to observe the model detection efficiency. The model with 6 predictors (AUC = 0.707) had reduced power compared to the model with 7 predictors, but by including fewer variables before surgery, it achieved better predictive power than the predictive model established by Fan et al.[14] Therefore, these two nomograms could identify POAF as early as possible.

Studies have shown that the operative time is associated with POAF, and a longer operative time may lead to more severe atrial ischemia and inflammation, which is more likely to induce POAF [18]. Off-pump theoretically reduces the inflammatory response and myocardial injury in patients [19], and this opinion was confirmed by Fan et al.[14] In this study, the usage rate of cardiopulmonary bypass in POAF group is higher than non-POAF group (11.3% v.s. 8.5%).However, there was no significant difference between the two groups. Some studies have pointed out that off-pump CABG is not superior to on-pump CABG in terms of decreasing inflammatory response and myocardial injury, and cannot completely avoid the occurrence of complications related to on-pump CABG [20]. The pros and cons of on-pump CABG and off-pump CABG are still a hot topic of discussion, and the specific differences are yet to be discussed and studied.

Age was an independent risk factor for POAF and was found to be the first among all predictors by the radar chart. Advanced age has been confirmed by many studies to be an independent risk factor for many cardiovascular diseases [21–23]. With age, aging is accompanied by cardiac fibrosis atrial structure remodels, resulting in reduced conduction [10, 24]. Hence, elderly patients have a high risk of POAF. The same, as the LAD increases in patients, it leads to structural remodeling of the heart [10]. A study has demonstrated that left atrial enlargement is associated with the occurrence of AF [25].

MAP is also an independent predictor of patients. Systolic hypertension is predominant in elderly patients, but it does not mean that elderly patients have higher mean blood pressure. MAP can directly reflect the preoperative arterial perfusion pressure. Studies have shown that higher MAP can improve tissue perfusion pressure and improve microcirculation in patients [26]. Higher MAP provides more adequate blood supply to the coronary arteries of the heart, thereby reducing the occurrence of AF. In univariate analysis, we found that LVEF was a potential risk factor, but independent predictor analysis showed that LVEF was not statistically different. Some studies have suggested that in patients with LVEF > 40%, there is no correlation between higher LVEF and better prognosis [27], and the impact of LVEF needs to be further studied.

BMI > 23 kg/m^2^ was a protective factor in the study, which has been noted in several heart disease studies as an obesity paradox [28, 29]. A study on BMI and prognosis of heart disease indicated that patients with a BMI between 23 kg/m^2^ and 35 kg/m^2^ had the lowest cardiovascular mortality [30]. Studies have mentioned a possible direct link between increased fat mass and cardiovascular protection [29], and secondly, some obesity-related risks possibly mitigated by effective management to mitigate accompanying risk factors [31], reducing the incidence of cardiovascular adverse events.

An RCT, including 200 patients, showed that administration of atorvastatin can reduce the occurrence of POAF and inhibit myocardium ischemic. This trial also indicated that statins reduce postoperative inflammatory factors released through stabilizing cell membrane ion channels [32]. Preoperative insulins use was found to be a protective factor in our study. It is perhaps related to the role of insulin in enhancing arterial compliance, increasing tissue blood flow, and increasing muscle microvascular blood volume [33]. A study shows that insulin has anti-inflammatory effects [34]. Ultimately, preoperative insulin use may improve postoperative myocardial ischemia and reduce inflammatory responses, thereby reducing POAF.

## Limitation

This study is a retrospective, single-center, small sample size study, which could be affected by some selection bias. Secondly, only patients with isolated CABG were included in this study, so the conclusions of this study cannot be directly used to guide the prevention of POAF in patients after valve surgery. The predictive power of the nomogram was moderate (AUC = 0.727 for the model with 7 predictors and AUC = 0.707 for the model with 6 predictors), and further better predictive models need to be proposed.

## Conclusions

We considered that Age, MAP, BMI > 23 kg/m^2^, LAD > 40 mm, Insulins, Statins, and intraoperative Operative Time > 4 h were independent predictors of AF after CABG. This nomogram might predict the individual probability of POAF and provide individualized protective treatment for patients.

## Data Availability

All data generated or analyzed during this study are included in this published article.
