# Correction: Nomogram predicts atrial fibrillation after coronary artery by pass grafting

**DOI:** 10.1186/s12872-022-02858-5

**Published:** 2022-09-22

**Authors:** Jingshuai Gong, Yangyan Wei, Qian Zhang, Jiwen Tang, Qing Chang

**Affiliations:** Department of Cardiovascular Surgery, The Affiliated Hospital of Qingdao University, 16 Jiangsu Road, Qingdao, 266003 China

## Correction to: BMC Cardiovascular Disorders (2022) 22:388 https://doi.org/10.1186/s12872-022-02824-1

Following publication of the original article [1], there is a formatting error in Tables 2 and 3. The corrected tables are shown below:

**Table 2:** Multivariable logistic regression analysis of 7 variables

Variables	Exp(B)	95% CI		P
Age, years	1.057	1.023	1.092	0.001
Operative time > 4 h, min	1.935	1.199	3.122	0.007
Insulins	0.544	0.324	0.913	0.021
Statins	0.551	0.326	0.93	0.026
LAD > 40, mm	1.879	1.062	3.323	0.03
MAP, mmHg	0.976	0.954	0.998	0.032
BMI > 23, kg/m^2^	0.549	0.308	0.979	0.042

**Table 3:** Multivariable logistic regression analysis of 6 variables

Variables	Exp(B)	95% CI		P
Age, years	1.055	1.021	1.089	0.001
Insulin	0.538	0.322	0.898	0.018
Statin	0.546	0.326	0.915	0.022
MAP, mmHg	0.977	0.955	0.999	0.037
LAD > 40, mm	1.774	1.012	3.111	0.045
BMI > 23, kg/m^2^	0.56	0.317	0.99	0.046

The original article has been corrected.

